# Adolescents with metabolic syndrome have a history of low aerobic fitness and physical activity levels

**DOI:** 10.1186/1476-5918-7-5

**Published:** 2008-04-04

**Authors:** Robert G McMurray, Shrikant I Bangdiwala, Joanne S Harrell, Leila D Amorim

**Affiliations:** 1 Departments of Exercise & Sport Science, University of North Carolina, Chapel Hill, NC, USA; 2 Department of Biostatistics, University of North Carolina, Chapel Hill, NC, USA; 3 School of Nursing, University of North Carolina, Chapel Hill, NC, USA

## Abstract

**Purpose:**

Metabolic syndrome (MS) is a clustering of cardiovascular disease risk factors that identifies individuals with the highest risk for heart disease. Two factors that may influence the MS are physical activity and aerobic fitness. This study determined if adolescent with the MS had low levels of aerobic fitness and physical activity as children.

**Methods:**

This longitudinal, exploratory study had 389 participants: 51% girls, 84% Caucasian, 12% African American, 1% Hispanic, and 3% other races, from the State of North Carolina. Habitual physical activity (PA survey), aerobic fitness (VO_2_max), body mass index (BMI), blood pressure, and lipids obtained at 7–10 y of age were compared to their results obtained 7 y later at ages 14–17 y.

**Results:**

Eighteen adolescents (4.6%) developed 3 or more characteristics of the MS. Logistic regression, adjusting for BMI percentile, blood pressure, and cholesterol levels, found that adolescents with the MS were 6.08 (95%CI = 1.18–60.08) times more likely to have low aerobic fitness as children and 5.16 (95%CI = 1.06–49.66) times more likely to have low PA levels.

**Conclusion:**

Low levels of childhood physical activity and aerobic fitness are associated with the presence of the metabolic syndrome in adolescents. Thus, efforts need to begin early in childhood to increase exercise.

## Background

The metabolic syndrome (MS) is a clustering of cardiovascular disease risk factors that includes glucose intolerance, hypertension, elevated triglycerides, low HDL cholesterol, and obesity [1-4]. This clustering has been shown to occur in adults [1-6] and in adolescents [7-12]. Since many factors included in MS can develop at an early age, before adolescence, the ability to determine which youth are at high risk for MS could be beneficial for early prevention programs. Two modifiable lifestyle factors that have been associated with the MS in adolescences are obesity [13] and the lack of exercise [14,15].

In children, obesity appears to be strongly associated with the MS [8,10,11,13,16,17]. Several cross sectional studies have also reported that low physical activity levels, sedentary lifestyle and low aerobic fitness (aerobic power or VO_2_max) in youth are associated with the MS [14-16,18,19]; the association being stronger in adolescents than in preadolescents [16,19]. Although these cross-sectional associations exist, we presently do not know if low levels of physical activity and aerobic fitness during childhood are associated with the presence of the MS during adolescence. The results of Ventura et al, however, suggest that neither physical activity nor inactivity during childhood is related to MS, but fitness during early adolescence (determined by the 20-m shuttle run) is associated with MS [19]. Although Ventura et al. followed a small cohort of girls (n = 152; 13.8% with MS) from ages 5 to 13 years, to our knowledge no other study has examined the same youth during childhood and adolescence to see if a history of low PA and aerobic fitness during childhood is linked to the presence of the MS during mid-adolescence. Thus, this study explored the relationship between childhood aerobic fitness and physical activity levels and the presence of the MS during adolescence in a cohort of youth. Our hypothesis was that the low levels of PA and aerobic fitness in adolescents with the MS were evident during their childhood.

## Methods

### Study participants

The sample used for this study was obtained from 7–10 years children from the Cardiovascular Health in Children and Youth Study – CHIC, which included 2207 children [20]. The original sample was obtained from 21 elementary schools, half rural and half urban, located throughout the State of North Carolina. The data for this report were obtained from 1990–1996. From this initial sample, some data on 1150 children was available seven years later; however, only 389 adolescents were found to have complete data at both time points. Moving out of the school district, rather than refusal, was the reason for the majority of subjects lost at follow-up. Although this appears to be a small cohort, it is considerably larger and more diverse than the cohort in a previous longitudinal study [19]. Our cohort used in this study consisted of 177 (45.5%) girls and 212 (54.5%) boys, which was similar to the gender distribution of the entire initial CHIC sample (n = 2207). Regarding ethnicity, the sample used in this study was 11.6% African American youth and 84.3% Caucasians, with a low representation of other ethnicities (4.1%). This ethnic distribution was somewhat different from the entire sample; 19% African American, 76% Caucasian and ~4% other. Informed consent was obtained from the parents and informed assent was obtained from the child prior to participation in the study. These forms were previously approved by the University's IRB.

### Procedures

The data used for this analysis were collected at each school site. Physical measurements were taken by trained and certified research assistants, who were routinely checked for quality control. Age, sex, and ethnicity were self-reported. Height was measured to the nearest 0.1 cm using a stadiometer (Perspective Enterprises, Kalamazoo, MI, USA). Body mass was measured to the nearest 0.1 kg using either a balance beam scale (Detecto Scale, Model 439, Webb City, MO, USA) or an electronic scale (Scaletronix, White Plains, NY, USA). Percent body fat was estimated from triplicate measurements of triceps and subscapular skinfolds [21] and was used to compute aerobic fitness in units of fat free mass (ml/kg_FFM_/min). Blood pressure was obtained in triplicate after a 5 minute quiet seated rest, using a Hawksley Random Zero Sphygmomanometer (Hawksley & Sons Limited, Sussex, England) with at least 1 minute between measurements. The mean of the three measurements was used for analysis of systolic and fifth-phase Korotkoff diastolic blood pressures.

Physical activity was estimated in elementary school youth from a self report survey that simply asked what three activities they do most often. Pilot testing on 732 children indicated that the self-report survey was positively correlated with parents' estimates of children's physical activity levels (r~0.29); similar to other studies in which physical activity was estimated from proxy reports [22,23]. PA levels were estimated during the 7-yr follow-up using a habitual activity survey comprised of 28 sedentary, low, moderate and vigorous intensity activities common to North Carolina adolescents [24,25]. The instrument has acceptable reliability with moderate coefficient alpha (0.74) and a two-week test-retest correlation of 0.70 [24]. For both surveys, PA scores were developed using the MET values for each activity multiplied by the number of times a week each was reported. Predicted aerobic fitness (_p_VO_2_max) was also estimated at both time points from a multi-stage submaximal cycle ergometry test [26]. The cycle ergometry test was found to be equally accurate for both genders (r~0.80 with actual measurements of VO_2_max) and has a standard error of estimate of ~150 ml/min or 4 ml/kg/min.

Total cholesterol was measured in a non-fasting state, between 7 to 9 am, from a finger-stick when the children were in elementary school. Reflotrons^® ^(Boehringer-Mannheim Diagnostics, Indianapolis, IN, USA) were used to analyze the blood. The instruments completed routine internal and external quality control (QC) using a CDC standard and CLIA approved Clinical Laboratory. When the subjects were adolescents, venous blood samples were obtained between 7 and 9 am, after an overnight fast. The fast was verified and any youth not fasting was excluded. The samples were immediately centrifuged and the serum or plasma separated and placed on dry ice for shipment back to the chemistry laboratory. Total cholesterol, high-density lipoprotein cholesterol (HDL-C) and triglycerides were tested on fasting plasma samples using automated coupled-enzymatic procedures and a Hitachi 911 analyzer (Boehringer-Mannheim Corporation), by the same Laboratory that completed the QC for the Reflotron. The serum glucose levels were measured using a Johnson & Johnson 950 automated chemistry system (University of North Carolina Hospital Core Chemistry Lab).

### Statistical Analysis

Although there appears to be no single accepted definition of MS in youth, the criteria of Jolliffe and Janssen was applied, which uses age-and sex-specific criteria linking childhood characteristics with adult MS criteria [27]. The association between baseline PA or VO_2_max and the presence of the MS seven years later was explored using exact methods of logistic regression models, which accounts for small sample size [28]. The dependent variable was the presence or absence of MS at follow-up (age 14–17 y). The independent variables were levels of PA and VO_2_max expressed per kilogram of body weight (mL/kg/min), or in terms of fat free mass (mL/kg_FFM_/min). Since there are no prescribed thresholds for physical activity levels or aerobic fitness, the lowest and highest tertiles of these two variables were used in the analyses. Also included were body mass index (BMI ≥ 95^th ^vs. <95^th ^percentile), elevated systolic or diastolic blood pressure (≥ 95^th ^percentile for age & gender), and elevated total cholesterol (>200 mg/dL [5.2 mmol/L]); all obtained at ages 7–10 years. We recognize that BMI is one possible criterion for MS, but not the sole component. Thus, it was examined as a possible predictor. Collinearity diagnostics were computed on the independent variables and all were below levels indicating significant collinearity: CI < 14.

## Results

The baseline and 7-yr follow-up characteristics of the subjects grouped by the presence or absence of MS at follow-up are presented in Table 1. Seven years after entry into the study 18 youth (4.6%) had at least 3 of the characteristics of MS. At baseline, the MS group already had higher (p < 0.04) mean BMI, systolic blood pressure, and total cholesterol; lower PA levels and aerobic fitness (pVO_2_max) than the non-MS group. Seven years later the MS group had higher (p < 0.002) body mass, BMI, body fat, systolic blood pressure, total cholesterol, triglycerides, and glucose than the non-MS group. The MS group also had lower HDL cholesterol, PA levels and aerobic fitness.

**Table 1:** Baseline and 7 yr follow-up characteristics (mean ± SD) of the participants presented by the presence or absence of the metabolic syndrome (MS) as adolescents

	Baseline		Seven Years Later	
	No MS	MS	No MS	MS

Age (y)	8.6 ± 0.8	8.7 ± 0.7	15.2 ± 0.7	15.1 ± 0.7
Height (cm)	135 ± 6	136 ± 6	168 ± 5	170 ± 8
Body Mass (kg)	32.3 ± 7.2	35.7 ± 6.2	63.7 ± 12.9	83.9 ± 12.9**
BMI (kg/m^2^)	17.6 ± 2.8	19.3 ± 2.0*	22.4 ± 3.9	29.0 ± 4.3**
Body Fat (%)	20.4 ± 7.4	26.0 ± 7.8*	24.5 ± 9.3*	36.6 ± 9.8*
BPsys (mmHg)	103 ± 10	108 ± 10*	114 ± 9	127 ± 10**
BPdia (mmHg)	67 ± 8	68 ± 9	68 ± 9	75 ± 11
Chol (mmol/L)	4.18 ± 0.75	4.60 ± 0.91*	3.77 ± 0.64	4.48 ± 0.78**
PA Score	67 ± 29	49 ± 20*	156 ± 69	122 ± 48**
VO_2_max (ml/kg/min)	44.3 ± 9.0	35.8 ± 11.6*	38.9 ± 10.2	29.7 ± 10.4**
VO_2_max (ml/kg_FFM_/min)	55.4 ± 9.7	48.4 ± 14.5	51.2 ± 10.1	46.9 ± 10.4
HDL-C (mmol/L)	----	----	1.23 ± 0.26	1.01 ± 0.20**
Trig (mmol/L)	----	----	0.86 ± 0.46	1.57 ± 0.7**
Glucose (mmol/L)	----	----	4.77 ± 0.42	5.14 ± 0.67**

* p < 0.04 MS vs. No MS at Baseline

** p < 0.002 MS vs. No MS at seven-years follow-up

The results of the exact logistic regression to determine the association between childhood aerobic fitness or PA and the presence of MS during adolescence are presented in Table 2. Children with low predicted aerobic fitness, expressed in mL/kg/min, were 5.5 – 6 times more likely to have MS as an adolescent. Similarly, children with low levels of PA were 5 times more likely to have MS as an adolescent. When childhood aerobic fitness was expressed in terms of fat free mass, the odds ratio for MS during adolescence comparing the low vs. high VO_2_max was not significant (p < 0.07); however, when the low aerobic fitness was compared to the moderate, the odds ratio was significant (p < 0.03).

**Table 2:** Results of the logistic regression presenting the relative risk for the association between the presence of the MS during adolescence and childhood aerobic fitness and physical activity levels.^1^

Childhood characteristic	Odds Ratio	95% Confidence Limits		p-value
		Lower	Upper	
pVO_2_max (mL/kg/min)				
Low vs. High	6.091	1.184	60.296	0.026
Low vs. Moderate	5.582	1.152	53.755	0.028
Physical Activity Level				
Low vs. High	5.115	1.054	49.131	0.041
Low vs.Moderate	2.265	0.682	8.757	0.220
pVO_2_max (mL/kg_FFM_/min)				
Low vs. High	3.642	0.932	20.826	0.067
Low vs. Moderate	5.706	1.197	54.485	0.023

pVO_2_max: Low, moderate and high tertiles

PA Score: Low, moderate and high tertiles

^1 ^Models adjusted for sex, elevated BMI, blood pressure, and cholesterol.

BMI: Above sex and age specific 95^th ^percentile

BP: Either systolic or diastolic >95^th ^percentile for gender and age Cholesterol: > 5.18 mmol/L

## Discussion

The proportion of adolescents in our sample that developed the metabolic syndrome was 4.6%. This proportion appears reasonable as it is within the range reported in the review manuscript of De Ferranti and Osganian for NHANES II & III adolescent population studies: 4.2–9.2% [13]. The authors noted that the rates of MS varied by definition and ethnicity. The results of our study showed that adolescents with the MS had low levels of physical activity and aerobic fitness as children. These associations appear to be quite respectable, as the odds ratios (~5–6 times greater) suggest good ability to separate true and false positive factions [29]. While studies have tracked cardiovascular disease risk factors separately (i.e. Ronnemaa et al. insulin [30]; Twisk et al. lipid profiles [31]; McMurray et al. [25] physical activity and aerobic fitness), the longitudinal relationship between exercise and MS has been minimally explored.

### Aerobic fitness and metabolic syndrome

Adolescents with MS were 6 times more likely to have a predicted VO_2_max of <37 ml/kg/min for girls and <43 ml/kg/min for boys at age 7–10 years. Further analysis indicated that the adolescents that had the MS were 5 times more likely to have aerobic fitness levels below the median for 7–10 year olds (40.8 and 46.6 mL/kg/min for girls and boys, respectively). This association between aerobic fitness and MS has been previously noted in cross-sectional studies of children [12,14-16,18,19] and adults [6], even when adjusting for the presence of the other MS factors. Thus our data, obtained seven years apart, suggests a longitudinal relationship between the MS and aerobic fitness in youth.

The reason for the relationship is multi-focal. Previous studies have indicated a strong relationship between obesity and MS in youth [10-13,16,17]. Our estimate of aerobic fitness was predicted maximal aerobic capacity expressed per unit of body mass. Body mass includes both fat mass and lean tissue mass. Fat mass contributes to the energy expenditure, but not the energy production. Also, our data on the whole sample of 2207 youth indicated good correlations between body mass and body fat, r = 0.79 to 0.72. Thus, individuals with greater fat mass (and greater body mass) would have lower values for aerobic fitness. It may be that expressing aerobic fitness in terms of kg body mass accounts for both aerobic fitness and fatness. Therefore, we also estimated aerobic fitness expressed *per kilogram fat free mass *and found that low levels of aerobic fitness, was still marginally associated with MS (Table 2). These results suggest the association of MS with aerobic fitness may be independent of fat mass. The improved metabolic capacity and insulin sensitivity of the "fit or trained" muscle are likely the mechanisms.

Aerobic fitness has been independently associated with the risk factors of MS, blood pressure, cholesterol, and insulin, as well as inflammatory factors in both children and adults [32-34]. In our data, aerobic fitness correlated significantly with total cholesterol (r = -0.23; p < 0.0001) and systolic blood pressure (r = -0.269; p < 0.0001). In addition, low aerobic fitness has been shown to be related to increased inflammatory markers in children and adolescents [33] and both cardiovascular disease and obesity have been characterized as low-level inflammatory diseases [35]. Thus, it follows that a relationship between MS and low aerobic fitness should exist. Genetics may also be a significant factor. Aerobic fitness also has a strong genetic component, accounting for 25–60% of the total phenotype variability [36]. The genetic components appear to exist for fat metabolism, fat deposition and body mass index, all important factors for the development of MS. Regardless of the reasons for the association of MS and the risk factors, our results extend the findings of these previous studies to show aerobic fitness during childhood is a strong indicator for the later development of MS during adolescence.

### Physical activity and metabolic syndrome

We found that adolescents with the MS were five times more likely to have low physical activity levels as children. Furthermore, our mean PA data suggests that in those youth who have the MS, low PA levels can persist from childhood into adolescence. Data in children and adolescents on the influence of PA levels and MS are sparse. Ravaji et al. found no influence of physical activity or inactivity at adolescence on the development of MS as a young adult, three years later [37]. In contrast, Kang et al. concluded that physical activity, particularly of a vigorous nature, significantly improves the MS profile of obese adolescents [38]. Some, but not all cross-sectional studies of children have found associations between MS and low levels of PA [14-16]; thus, there is some support for a positive influence of physical activity on MS.

As with aerobic fitness, the relationship between MS and PA levels makes sense when one considers the well-established dose-response relationship of physical activity and cardiovascular disease risk factors [39]. In addition, participation in regular PA, particularly of a vigorous nature, has the potential to increase aerobic fitness in children [25]. We noted a low, but significant, correlation between PA score and aerobic fitness in our subjects (r = 0.13; p = 0.01); however, the correlations between vigorous PA and aerobic fitness was stronger (r = 0.34; p = 0.0001). Finally, physical activity not only has the capacity to increase fitness, but also increases energy expenditure, which influences weight status. Abbott et al. noted subjects reporting the most vigorous PA had the lowest percentages of body fat and BMIs [40]. Hence, there are many ways that PA could impact the MS.

### Strengths and limitations

Our study is the first to report the importance of childhood PA levels and aerobic fitness on the presence of MS during adolescence. The strength of the study is related to the seven-year period between the baseline and follow-up measurements in the cohort. To our knowledge, only one other study [19] has presented longitudinal data on MS and that study included only a small cohort of girls (n = 152). One of the limitations of our study is the low number of subjects that developed MS. In our defense, de Ferranti and Osganian have summarized the epidemiology research on adolescent MS (NHANES II & III), noting that the rates of MS varied by definition and ethnicity, but was approximately 4.2–9.2% of the overall adolescent population [13]. Thus, our proportion (4.6%) is reasonably similar to their larger population sample. In addition, we employed the 'exact methods' of logistic regression used by SAS to adjust for small samples [28]. Another limitation of the study was the use of the PA survey to estimate habitual PA levels. Surveys are notoriously problematic. However, we had to design an instrument that could be used in a large sample of youth and was developmentally appropriate for elementary school children and high school students in North Carolina. The instrument has been shown to track PA levels over time [25] and thus, was appropriate for this study.

## Conclusion

The results of this study show that adolescents with the metabolic syndrome had low levels of aerobic fitness and low levels of physical activity as children; suggesting that low aerobic fitness and PA levels may be risk factors for the early development of multiple metabolic syndrome as an adolescent. The results also suggest that in children, to reduce the risk of developing the MA, physical activity should be of sufficient intensity to increase the aerobic fitness.

## Competing interests

The author(s) declare that they have no competing interests.

## Authors' contributions

RGM was responsible for the initial training of the research assistants to insure quality control, the design of the study, and was primary author of the manuscript. SIB designed the data management system, the analytical approach to the data and revising the manuscript. JSH was responsible for obtaining the data, training the research assistants, and revising the manuscript. LDA was responsible for the data analyses, revising and approving the final draft of the manuscript. All authors read and approved the final version of the manuscript
